# An Indoor Positioning System Based on Static Objects in Large Indoor Scenes by Using Smartphone Cameras

**DOI:** 10.3390/s18072229

**Published:** 2018-07-11

**Authors:** Aoran Xiao, Ruizhi Chen, Deren Li, Yujin Chen, Dewen Wu

**Affiliations:** 1 State Key Laboratory of Information Engineering in Surveying, Mapping and Remote Sensing, Wuhan University, Wuhan 430079, China; xiaoaoran@whu.edu.cn (A.X.); wudewen@whu.edu.cn (D.W.); 2 Collaborative Innovation Center of Geospatial Technology, Wuhan University, Wuhan 430079, China; 3 School of Geodesy and Geomatics, Wuhan University, Wuhan 430079, China; yujin.chen@whu.edu.cn

**Keywords:** indoor positioning, smartphone, large indoor scene, computer vision, deep learning

## Abstract

The demand for location-based services (LBS) in large indoor spaces, such as airports, shopping malls, museums and libraries, has been increasing in recent years. However, there is still no fully applicable solution for indoor positioning and navigation like Global Navigation Satellite System (GNSS) solutions in outdoor environments. Positioning in indoor scenes by using smartphone cameras has its own advantages: no additional needed infrastructure, low cost and a large potential market due to the popularity of smartphones, etc. However, existing methods or systems based on smartphone cameras and visual algorithms have their own limitations when implemented in relatively large indoor spaces. To deal with this problem, we designed an indoor positioning system to locate users in large indoor scenes. The system uses common static objects as references, e.g., doors and windows, to locate users. By using smartphone cameras, our proposed system is able to detect static objects in large indoor spaces and then calculate the smartphones’ position to locate users. The system integrates algorithms of deep learning and computer vision. Its cost is low because it does not require additional infrastructure. Experiments in an art museum with a complicated visual environment suggest that this method is able to achieve positioning accuracy within 1 m.

## 1. Introduction

It seems obvious for us to conclude that human beings as well as most of animals locate themselves by visual perception. According to our own experiences, people observe the environment surrounding them intentionally or unintentionally, and “draw” a rough map in their mind. The winners of The Nobel Prize in Physiology or Medicine 2014 proved this idea: O'Keefe et al. [1,2] discovered that some “place cells”, which are a type of nerve cells in particular area of the brain, were always activated if a rat was located at a particular place in a room. Other place cells were also activated if the rat moved to different places. O’Keefe concluded that the room map in the mind is formed by these place cells. In 2005, May-Britt and Edvard Moser [3] made a further discovery. They found another important component of location system in brain. Another type of nerve cell, which they named “grid cells”, that can create a coordinate system to realize precise positioning and find accurate paths. Their subsequent research [4] indicated how place cells and grid cells are able to determine position and allow one to navigate.

Inspired by this interesting discovery, we propose an indoor positioning system. We used smartphone cameras as data acquisition devices to capture visual information for location. Considering that specific indoor surroundings, including objects and layouts, activate place cells in the rat’s brain, we decided to use particular objects in the rooms for positioning. In an application, objects used as positioning references should be common and immovable, such as doors and windows. These objects are widely distributed (many of which are necessary for indoor scenes) and remain still and well-preserved for a long time. As their locations are known in plan maps, they become ideal reference sources for positioning. In this paper, we called them “static objects”. Besides, large indoor scenes such as libraries, airports and museums, provide a wide and stable vision condition for visual algorithms of the system. Traditional vision-based indoor positioning methods have their own limits when implementing in this kind of relatively large indoor environments (See Section 5.2. Evaluation). Thus, we decided to use the system to locate people in large indoor scenes.

In our previous research, Wu. et al. [5] compared the positioning accuracy between human brains and a visual algorithm was proposed, which proved that the indoor visual positioning method via smartphone outperforms human brains. They experimented with the positioning algorithm in several places including libraries and an office to prove the robustness. In this paper, we improved the method to a whole system, realizing positioning in large indoor spaces in our application.

The system proposed in this paper aims to locate smartphones (users) via static objects. Static objects in image are detected and identified firstly. Then the positions of users are calculated as output. The main contribution of our work can be summarized as follows:

(1) We propose an indoor positioning system by using smartphone cameras, which is designed for large indoor scenes. Previous studies of indoor positioning based on smartphone cameras have their own shortcomings in such large indoor scenes. The system integrates computer vision (CV) and deep learning (DL) algorithms. Common static objects (such as doors and windows) in the indoor scene are used as references for locating purposes, making our method general, and easy to replicate.
(2) We tested our system in a large indoor space with a complicated field of vision—an art museum. Experiments indicated that our method is able to achieve a positioning accuracy within 1 m in such circumstances.
(3) Our method is low-cost, as developers only need to take several photos to the static objects as a sample collection, without any additional infrastructure. It is also easily operated using monocular photography, which means users don’t have to photograph scenes from multiple angles or take a video.

The rest of the paper is organized as follows: Section 2 reviews related works. Details about the method are demonstrated in Section 3. Experiments with performance evaluation are presented in Section 4. Section 5 is the discussion and Section 6 is the conclusions.

## 2. Related Works

Despite of the increasing demand for indoor location-based services, there is still no persuasive solution for indoor location and navigation like Global Navigation Satellite System (GNSS) for outdoor environments, as GNSS signals are too weak to penetrate into walls. Also, the complex spatial topology and RF transmission make localization in indoor environments very complicated.

Recent years have witnessed many studies on indoor positioning, especially by means of smartphones for their widespread use and development. Equipped with various sensors and supporting rich RF signals, smartphones hence can be located by various means, which can be divided into three categories [6]: (1) GNSS signal receiver, including GPS, BDS, GLONASS and Galileo; (2) Built-in sensors in smartphones, such as accelerometers, gyroscopes, magnetometers, barometers, lighter sensors, microphones, loudspeakers and cameras, etc.; (3) RF signals like Wi-Fi, Bluetooth and cellular wireless communication signal, etc. Except for the GNSS signal receiver, all of other sensors and RF signals are not designed for positioning purposes, but they can be used to calculate indoor locations via different principles and algorithms. Among them, the establishment of fingerprinting of Wi-Fi [7,8,9,10,11], geomagnetism [12] or Bluetooth [13,14,15,16] are popular approaches due to their effectiveness and independence from infrastructure. These methods can reach positioning accuracies of 2~5 m but are easily interfered by changing environments and nearby human bodies. Besides, the fingerprinting database has to be updated every few months, which is inconvenient for developers. He et al. [17] combined the strengths of trilateration and fingerprinting to form an accurate and effective indoor localization framework. The Bluetooth antenna array [18] can achieve a higher location accuracy but has limitations such as high cost and short working distance. Cellular technology [19,20] has great potential in indoor positioning because cellular signals are widespread, but the positioning error of these kind of methods is relatively large. Infrared technology [21] and ultrasonic waves [22,23] can achieve higher indoor positioning accuracy, with the limitation of needing additional infrastructure. Since all of these methods have their own advantages and disadvantages, multi-sensor fusion approaches have been researched by many people to take advantage of different methods. Kim et al. [24] combined magnetic signals, Wi-Fi signals and cellular signals to realize indoor location. Jeon et al. [25] proposed a method integrating Bluetooth RSSI with an accelerometer and a barometer on smartphones to reach higher positioning accuracy compared with the approach without Bluetooth RSSI. Chen et al. [26] integrated a typical Wi-Fi indoor positioning system with a PDR system and achieved a better positioning performance than the PDR system or Wi-Fi positioning system alone. Li et al. [27] proposed a dead-reckoning (DR)/Wi-Fi fingerprinting/magnetic matching (MM) integration structure. The structure uses consumers’ portable devices with off-the-shelf sensors and existing Wi-Fi infrastructures to raise positioning accuracy. Liu et al. [28] fused multiple sensors, including cameras, Wi-Fi and inertial sensors, and used a deep learning method to realize indoor localization. Becker et al. [29] used vision images for classification to recognize corridors, with existing wireless LAN access points in corresponding corridor to realize positioning. Gao et al. [30,31] designed a method which combined camera and gyroscope in smartphone to realize indoor positioning with accuracy of 2 m–8 m. In their method, at least three smartphone photos are photographed in each test place to capture references points (such as store logos) that provide positioning information, and gyroscope records angle information at the same time. After that, the position can be calculated via trilateration.

In addition, the vision-based indoor positioning problem has always been a hotspot issue of research in the last decades. In 1998, the vision technology group of Microsoft discussed an easy-living life in the future [32], and one of the core technologies was to locate people in house by using several video surveillance techniques. After that, methods of indoor positioning based on vision kept developing, and they can be roughly divided into three categories [33]. The first category uses references from building models. These kinds of methods detect objects in images and match them with those in the building database. Hile and Borriello [34] compared the images with the floor plan of the building to locate smartphones. Kohoutek et al. [35] detected specific objects in cloud points obtained by a range imaging camera, and compared them with the digital spatial-semantic interior building model CityGML to determine location and orientation. The second category of visual positioning methods are based on images. These approaches mainly compare similarities among testing images and reference images captured in offline phase and output the location of the reference image with the highest score. Kim and Jun [36] matched the current view of a camera with image sequences stored in a database. Their method is designed for augmented reality applications for extra information. Werner et al. [37] estimated position and orientation by using reference images and location information acquired from the pre-built database. They designed an algorithm to estimate distance through the ratio of matched pixel distance to measure viewpoint-to-image distance. Möller et al. [38] designed an mobile indoor navigation system combined interfaces of Virtual Reality (VR) and Augmented Reality (AR) elements. The last category is utilizing deployed coded targets, including concentric rings, barcodes or patterns consisting of colored dots, etc. Mulloni et al. [39] pasted the barcode in different places, so cameras can capture these marks to get location as well as other information. ByteLight company created a special LED light with specific frequency (represent different position information), which can be captured by cameras instead of human eyes [40]. In addition to these systems and methods, visual gyro [41] and visual odometer technology [42] are also used as visual positioning. The algorithm of vision positioning is more complex, larger computation and higher power consumption than other methods. However, with further improvement of smartphone performance, this kind of methods is expected to further popularize in the future.

## 3. System and Methodology

In this section, a system overview is presented at first. Then key modules are illustrated with process of the system.

### 3.1. System Overview

**Figure 1:**
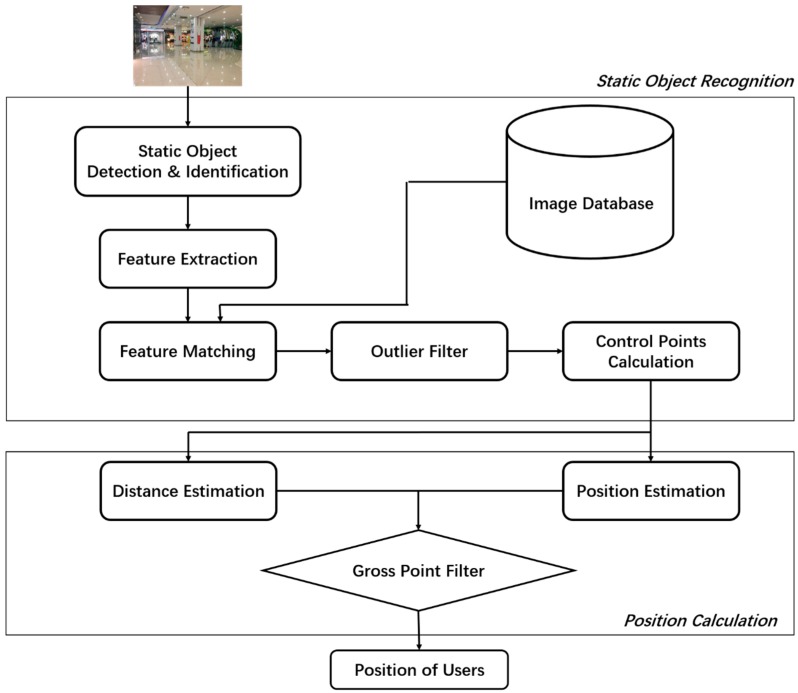
System flow diagram.

The main idea of our system is to use smartphone images to locate users via static objects in large indoor scenes, and the system flow diagram is shown as Figure 1. The proposed indoor positioning system consists of two parts: static objects recognition and position calculation. The static objects recognition aims to detect and identify static objects in images, and then determine coordinates of control points for calculating users’ location (Section 3.1). The position calculation includes position estimation, distance estimation and a filter screening gross points and output users’ position (Section 3.2).

The current version of our system is web-based. After the smartphone photographs test images, a desktop as server will implement the rest of algorithms and return the results.

### 3.2. Static Objects Recognition

#### 3.2.1. Static Object Detection & Identification

When users take a photo as input, the first task of the system is to detect static objects in images and recognize their unique identities. This is a key module of the system, outputting boundaries and identities of static objects in images. The boundaries of static objects in image influence the performance of feature extraction and matching in the following procedures, and identities of static objects are the key to find corresponding attributes in database, such as room number, pixel coordinates and objects coordinates of control points, etc.

**Figure 2:**
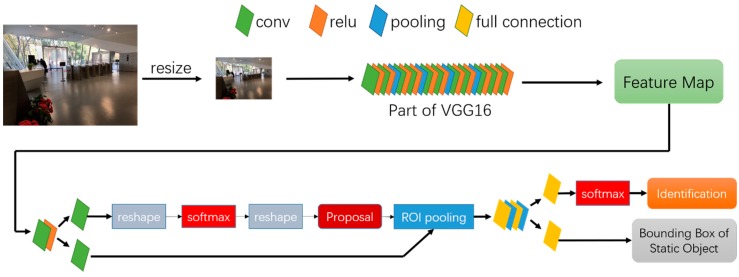
Faster-RCNN network for static objects detection and identification.

In this paper, we implement Faster-RCNN algorithm [43] for this task. Faster-RCNN integrates region proposal, feature extraction, classification and rectangle-refine into one end-to-end network, which greatly reduce the amount of calculation and speed up the detection process. Smartphone images are firstly zoomed into a fixed size, then those fixed-size images are send to the network. Just as Figure 2 illustrates, at the beginning of the network there are 13 conv layers, 13 relu layers and four pooling layers. This combination of different layers is actually a part of VGG16 network [44], which is a famous network in image classification, realizing feature extraction for smartphone images; Then a Region Proposal Network (RPN) generates foreground anchors as well as bounding box regression bias to calculate proposals from these features; ROI pooling layers use proposals to extract proposal feature for subsequent fully convolutional network and softmax network to classify proposals. The whole network is trained on the basis of the convergence of loss function as below:

(1) $$\mathrm{Loss}=\frac{1}{N_{cls}}\sum_{i}L_{cls}\left(p_{i},p_{i}^{*}\right)+\frac{1}{N_{reg}}\sum_{i}p_{i}^{*}L_{reg}\left(t_{i},t_{i}^{*}\right)$$

Here, i is the index of anchor; p represents prediction probability for classification of foreground anchor (i.e., static object) and t is the outer rectangle of predicted target. $p^{*}$ and $t^{*}$ represent the corresponding ground truth of p and t respectively. $N_{cls}$ and $N_{reg}$ are numbers of anchors and outer rectangles. $L_{cls}\left(\cdot \right)$ and $L_{reg}\left(\cdot \right)$ run subtraction.

**Figure 3:**
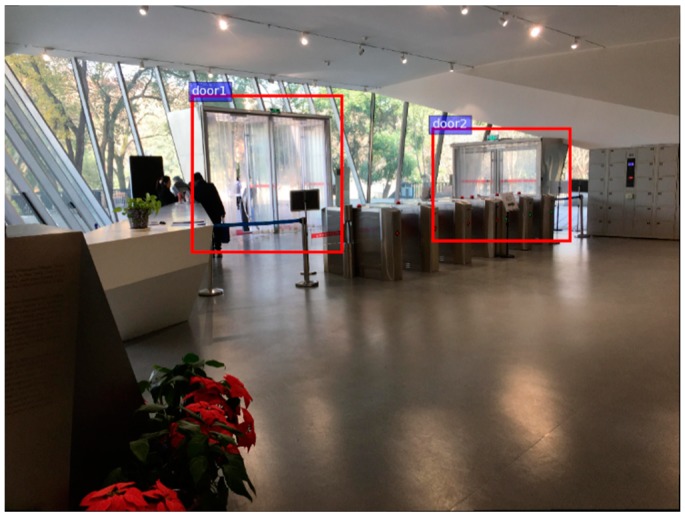
An example of output (right) in process of Static Object Detection & identification.

In order to improve performance and robustness of the system, the whole network shall be retrained in offline phase. Photos of static objects photographed from various angles at different distances are used for training images. After training customized network, the system outputs outer rectangles and identities of static objects appeared in images (as Figure 3).

#### 3.2.2. Obtaining Control Points Coordinates

We define “control points” as those physical feature points on static objects with accurately surveyed coordinate location and can be identified relatively easy. By building relationship between pixel coordinates in image and corresponding space coordinates of control points (Collinear Equation Model [45]), the position of the smartphone can then be obtained. Thus, the key problem is to find the corresponding pixel coordinate of control points in test images. Here is our strategy: In the offline phase, images of static objects are photographed and stored in dataset, called the “reference images”. Pixel coordinates of control points in these images are measured and recorded. In the online phase, after detecting and identifying static objects in test images, feature points in testing image and corresponding reference images are extracted. Then the feature matching algorithm is implemented to get enough homonymy feature points, which is used to calculate homographic matrix in next step. The homographic matrix represents the mapping relationship between pixels of testing image and reference image. Finally, the pixel coordinates of control points in testing image can be calculated from the homographic matrix and reference images coordinates of control points. The details of the algorithm are showed in Algorithm 1.
**Algorithm 1.** Obtaining Pixel Coordinates of Control Points in Test Images**Input**: image block of static objects from test image**Procedure**:  (1) Get reference image through identity of static object from database;  (2) Extract feature points for both test image block and reference image by SIFT operator [46];  (3) Perform feature matching to get homonymy feature point pairs;  (4) Employed RANSAC [47] to remove false matching points; the remaining matching points marked as $P_{test}$ for test image and $P_{ref}$ for reference image;  (5) Calculate homographic matrix $H_{homo}$ by solving formula below:
$$P_{Test}=H_{homo}\times P_{ref}$$  (6) Estimate pixel coordinates of control points in test images CPT as following formula, $CPT_{ref}$ is the set of pixel coordinates of control points in reference images:
$$CPT=H_{homo}\times CPT_{ref}$$**Output:**CPT

**Figure 4:**
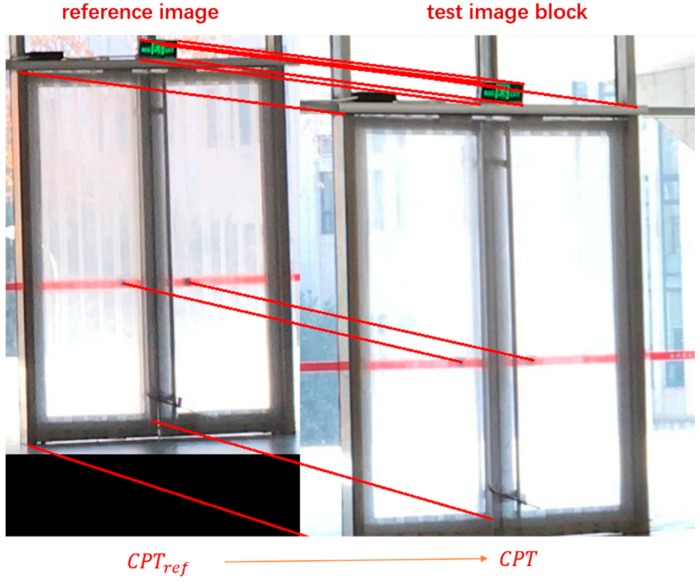
An example of output by Algorithm 1. The pixel coordinates of control points in test image are obtained from reference image.

Figure 4 shows an example of output by Algorithm 1. The pixel coordinates of control points in reference images are measured in the offline phase. The reason why we do not directly choose feature points from test image as output is that the specific control points may not belong to the set of feature points by SIFT when the texture of images are too complicated. Also, it is hard to design a robust and effective filter to screen the specific point from plenty of feature points. Algorithm 1 is a fast and effective approach instead.

### 3.3. Position Calculation

#### 3.3.1. Position Estimation

The geometric relation between control points in image and object space can be illustrated via collinear equation model as Equation (2), for pixel coordinate (x,y) and space coordinate (X,Y,Z) of the same control point, the geometric relation can be illustrated as below:

(2) $$\{\begin{array}{c} x-x_{0}=-f\frac{t_{11}\left(X-X_{O}\right)+t_{12}\left(Y-Y_{O}\right)+t_{13}\left(Z-Z_{O}\right)}{t_{31}\left(X-X_{O}\right)+t_{32}\left(Y-Y_{O}\right)+t_{33}\left(Z-Z_{O}\right)}\\ y-y_{0}=-f\frac{t_{21}\left(X-X_{O}\right)+t_{22}\left(Y-Y_{O}\right)+t_{23}\left(Z-Z_{O}\right)}{t_{31}\left(X-X_{O}\right)+t_{32}\left(Y-Y_{O}\right)+t_{33}\left(Z-Z_{O}\right)} \end{array}$$

**Figure 5:**
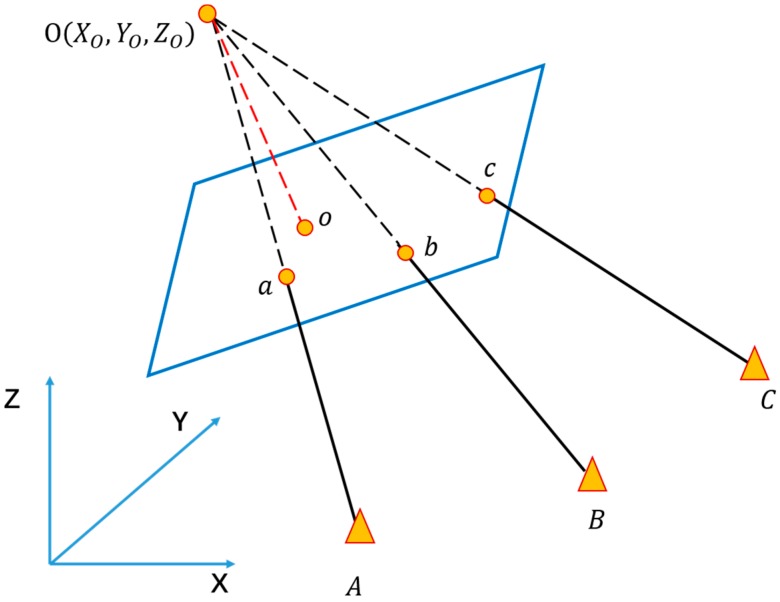
Principal of Position Estimation. $o\left(x_{0},y_{0}\right)$ is the principal point of camera image, and the length of Oo is the focal length f. By calculating collinear equation models of control point pairs, *A*, *B*, *C* and *a*, *b*, *c*, the positioning of smartphone $O\left(X_{O},Y_{O},Z_{O}\right)$ can be obtained.

In this formula, $\left(x_{0},y_{0},f\right)$ are the intrinsic parameters of the camera, which can be measured by camera calibration offline [48]. $\left(X_{O},Y_{O},Z_{O}\right)$ are the space coordinates of the smartphone camera, i.e., the position of user. $t_{ij}\left(i,j=1,2,3\right)$ are nine directional cosines related to the exterior orientation of smartphone. Hence, as long as more than three control points are offered (including pixel coordinates and space coordinates, which are the result of CPT from last step, Section 3.2.2), such as (A,a), (B,b) and (C,c) in Figure 5, the position can be calculated through this model. The system outputs estimated position $\left(X_{O}^{*},Y_{O}^{*},Z_{O}^{*}\right)$ by an iterative process.

#### 3.3.2. Distance Estimation

In order to avoid gross error for the final position, distance estimation is then implemented to check the output of collinear equation model.

**Figure 6:**
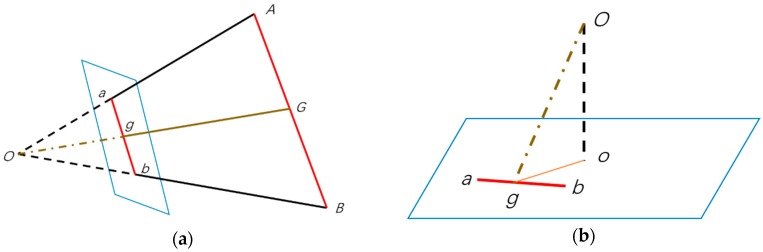
Principal of Distance Estimation. (**a**) Is geometric relation between smartphone camera and control points on static object; (**b**) is interior geometric relation of smartphone camera.

The principle of distance estimation can be illustrated as Figure 6a: A and B are two control points. The parallelogram in blue represents image plane; a and b are corresponding pixels of control points in image. G and g are midpoints of line AB and ab respectively. O is the focal point, which is also the position of the smartphone. The distance between the smartphone and a static object can be simplified as the length of line OG, which can be estimated by following formula:

(3) $$OG=\frac{Og}{ab}\times AB=d_{r}$$

The length of Og can be calculated as Figure 6b: o is principal point of camera image, line Oo is the focal length f. Thus:

(4) $$Og=\sqrt{{\left(Oo\right)}^{2}+{\left(og\right)}^{2}}$$

In addition, since the position of smartphone $O=\left(X_{O}^{*},Y_{O}^{*},Z_{O}^{*}\right)$ has been estimated in previous, we can calculate the distance OG directly and marked it as $d_{e}$. Then the controlled error γ as following are used to screen out gross error. If γ is less than the threshold, the estimated position is acceptable as final system output:

(5) $$\gamma =\|d_{r}-d_{e}\|$$

## 4. Experiments

In this section, details of experiments are represented. We tested our system in a large indoor space with a relatively complicated environment and compared the positioning results with the ground truth.

### 4.1. Experiment Setup

**Figure 7:**
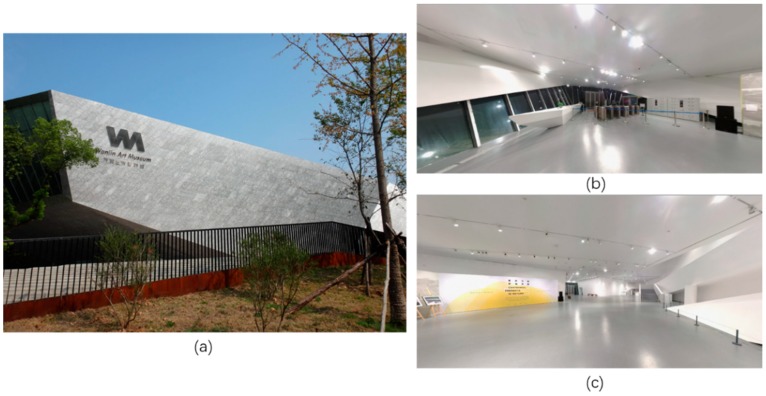
Test environment of the proposed system, i.e., Wanlin Art Museum. (**a**) is outside look of the art museum. (**b**,**c**) are photos of inside look on the first floor.

**Figure 8:**
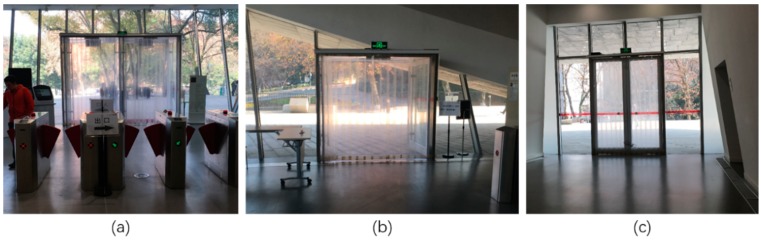
Three glass doors as static objects in the experiment. (**a**–**c**) are “door1”, “door2” and “door3” respectively.

The experiment was conducted on the first floor of Wanlin Art Museum in Wuhan University. The museum has about 8400 m^2^ building area, and its first floor has more than 1000 m^2^ with an open field and stable illumination, which is a typical large indoor scene (Figure 7). In order to verify the effectiveness, static objects shall be common and easy-to-catch in image. There are three glass doors in the experimental site, all of which can be seen at any place of the room (Figure 8). Two doors (identified as “door1” and “door2”) are on the south of the museum, and “door3” locates in the north. We chose these three glass doors as static objects and used them for locating.

**Figure 9:**
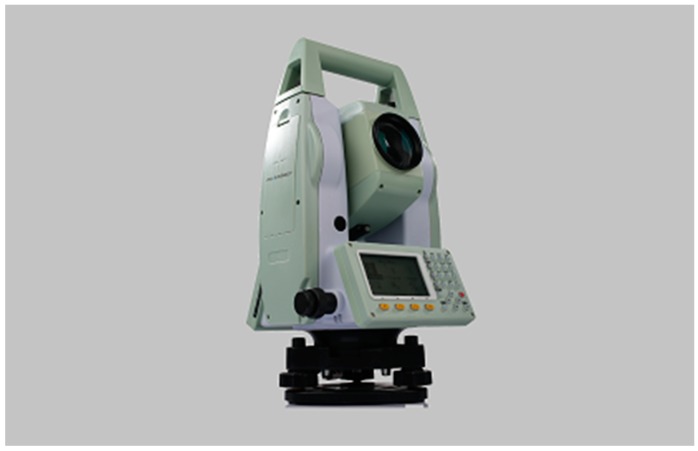
Hi-Target ZTS-420R Total Station is used for measuring space coordinates of control points and ground truth of test points.

We used an iPhone 6S smartphone to take images in both offline and online phases, including training images for network, reference images and test images. Other procedures of the system were conducted in a computer with Titan Xp graphics card, for a purely web-based solution. In this case, the batteries of smartphones do not consume much power. The ground truth of space coordinates for all control points are measured by a Hi-Target ZTS-420R Total Station (Hi-Target Surveying Instrument Co. Ltd, Guangzhou, China) (Figure 9), with 2 mm positioning error for every 1000 m distance.

We randomly selected twelve places as test points and photographed plenty of static objects images. All the test points are distributed throughout the room evenly (Figure 12).

### 4.2. Performance of Static Object Recognition

In the phase of static object detection and identification, we did data augmentation for training images in order to prevent the network from overfitting and improve the success rate of static object detection. We randomly blocked 30% area of the static target area in the training image to simulate the actual situation that the static objects may be blocked by pedestrians or other things. There were 302 training images in total. We adopted the strategy of transfer learning, and retrained the networked by using training images on the basis of a model trained by ImageNet [49]. Using the cross-validation method, we randomly selected 50% of the images for training, 25% for testing, and 25% for validation. The Accurate Precision (AP) of the detection and identification is shown in Table 1.

**Figure 10:**
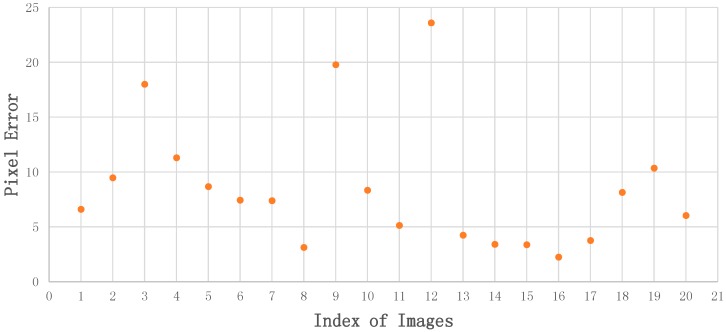
Accuracy of control points coordinates in test images. The horizontal axis is the index of test images; The vertical axis is pixel error between obtained pixel coordinates of control points and ground truth.

The accuracy of coordinates of control points in test image is determined by the homographic matrix $H_{homo}$, which determines the final positioning accuracy to a large extend. We manually measured and recorded the ground truth of control points’ pixels in test images (with 2~3 pixels error) and compared them with the calculation results CPT. The errors of pixel coordinates for matching, as showed in Figure 10, mostly fall within ten pixels, which we considered as acceptable.

The size of images photographed by iPhone 6S is 3024 × 4032 pixels. For all of test images with such size, time cost in static object recognition phase are about 0.3 s.

### 4.3. Positioning Results and Analysis

**Figure 11:**
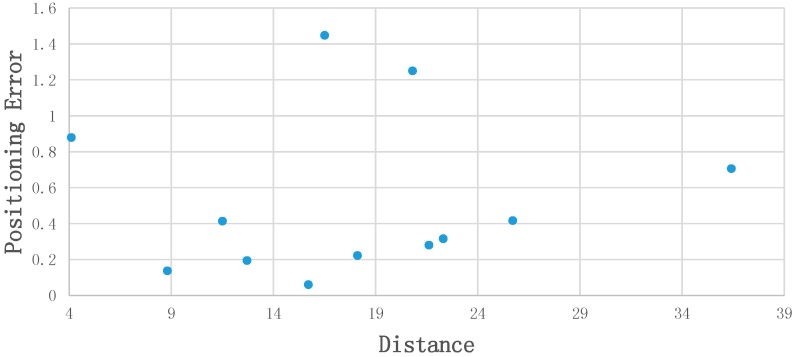
Relation between position error and distance.

This part demonstrates positioning results. We evaluated the accuracy through Euclidean distance of calculated position and ground truth position. Figure 11 illustrates the relationship of distance (user-static object) and positioning error. Within the range of 40 m, there is no significant correlation between distance and positioning precision. All of the test points achieved an accuracy of 1.5 m, and most of them are within 1 m.

**Figure 12:**
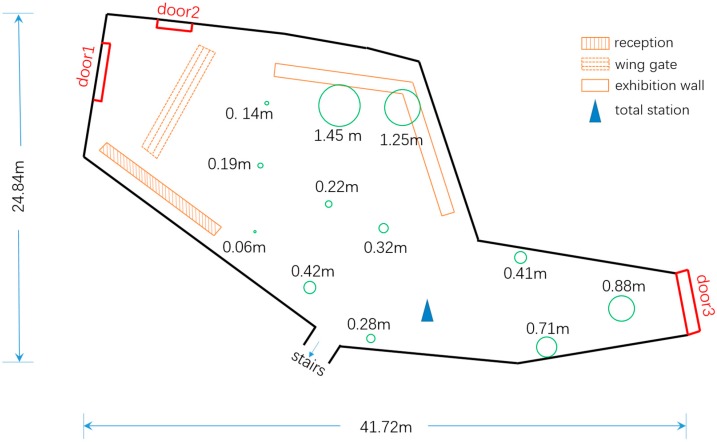
Plan map of experimental site and positioning results.

Figure 12 is the plan map of the experimental site. Green circles on the map are error boundaries, and centers of these circles represent test points. The words near circles such as “0.14 m” means that the positioning accuracy error of this test point is 0.14 m. Just as the figure shows, nearly all test points achieve accuracy within 1 m, except for two test points, which is caused by unreasonable distribution of control points (see Section 5. Discussion). From the plan map we can see that our system has ability to locate smartphone within accuracy of 1 m in such a large indoor scene.

## 5. Discussion

Large indoor spaces with wide field of vision and stable illumination (such as museums, malls and airports) provide applicable environments for visual positioning. Hence, we use common static objects in these spaces as references to locate smartphone camera via visual algorithms. Our experiment in an art museum suggests that our system is able to achieve positioning accuracy within 1 m even that the experimental environment is complicated in vision.

### 5.1. Experimental Difficulties and Criteria for Choosing Static Objects

The static objects (doors) we chose in experimental site are actually complicated to process for a visual algorithm, because they are made of glasses. Since glass doors are transparent, their texture in image is depends on the outside environment, which can change due to many factors, like weather, time, season, illuminance and shooting angles, etc. This characteristic limits performance of feature extraction and feature matching. In this case, the strategy that designing a feature filter to get homonymy points of feature points from reference images directly, as the final control points in test image, is not robust: At first, pixels of control points in test images may not be extracted as feature points. In such condition, choosing the nearest neighbor feature points in test image increases pixel coordinate error. Secondly, it is hard to design a robust feature filter to select the correct one from huge amount of feature points when facing changeable image texture. Thus, we designed the strategy to get control points in test images from both homographic matrix $H_{homo}$ and control points in reference images instead. By this strategy, the practicality of the system increases. However, in order to generate more feature points to increase accuracy of homographic matrix $H_{homo}$, static objects with non-transparent material will be better. Although we used doors as static objects in our experiments, anything can be set as static objects in indoor scenes, which increases the practicability of the method.

Besides, control points on static objects shall be chosen properly. Two test points with higher positioning accuracy error (1.45 m and 1.25 m respectively) resulted from improper distribution of control points. Only “door1” can be seen on these two places, and the control points in images were roughly distributed on a straight line. Other test points that have proper ‘observation condition’ reached ideal accuracy. Generally, control points shall be easy-to-capture in image, with characters different from neighboring regions, such as those with unique color or edge points, etc., and they shall be distributed evenly throughout the static objects. Further, static objects chosen as references cannot be too small, otherwise all control points will be distributed too close to output accurate positioning results.

### 5.2. Evaluation

Vision-based indoor positioning problem has always been a hot research issue in recent years. However, methods that are completely based on vision algorithms and conducted on smartphones are much less frequent, and each method has its own scope of application. Due to the low cost or even without the need for any infrastructures as well as the popularity of smartphones, we believe that this kind of method has a promising future. However, the methods or systems designed so far may have their own shortcomings when implemented in large indoor scenes. In the following, we will discuss and evaluate these state-of-art of purely visual indoor positioning methods or systems when performing in large indoor spaces, most of them are based on smartphone cameras as our system. We excluded methods that integrated smartphone camera with RF signals, such as Wi-Fi, Bluetooth, wireless LAN and so on, because our system uses vision algorithm only, and thus without any infrastructures.

The *Signpost* system designed by Mulloni et al. [39] detects unobtrusive fiduciary markers that contain position information in different places as the location of users. This strategy is easy to transplant due to the low cost and low computation power of cellphones, but users have to find the nearest marker as these tiny markers cannot be made large in size. This is inconvenient for people in large indoor spaces since they may have to move a long distance to find and scan a marker. Hile et al. [34] built a novel system to find precise locations and orient users by matching features extracted from smartphone images with relevant sections of a floorplan map. However, as this creative system is designed for environments like hallways, it may perform poorly in large indoor scenes, because the available features such as edge or corners are much less abundant in large spaces. The distance estimation algorithm by Werner et al. [37] is adopted by our system as Distance Estimation module (Section 3.3.2). This algorithm is able to calculate accurate distances between cameras and reference targets. However, since the trilateral location method requires at least three reference target positions as well as the corresponding distances to the camera (one distance for each image), this algorithm performs better in environments such as corridors with narrow width (requiring one target/distance only) than wide open indoor districts (require at least three targets/distances). Van Opdenbosch et al. [50] realized indoor positioning by image recognition. In order to achieve meter-accurate localization, they photographed images in every 1 m × 1 m grid cell, using 16 viewing angles for each spot. However, this strategy has low efficiency in larger rooms because the vast number of images in the dataset will result in a huge computation cost and increase the burden of smartphones and web servers. Kawaji et al. [51] realized indoor positioning in a large indoor space—a railway museum. They used omnidirectional panoramic images captured by an omnidirectional camera and supplemental images captured by digital cameras to build a dataset and matched the test digital camera images with reference images in the dataset to locate users. Since omnidirectional panoramic images are suitable for large scenes, this method is effective for localization in large indoor spaces. However, the output location of test images is the same as that of the omnidirectional panoramic images, which cannot achieve a relatively accurate position. Deretey et al. [52] proposed a method by matching camera images with 3D models of indoor scenes by a Simultaneous Localization and Mapping (SLAM) system to realize positioning. Although it requires a 3D model building process in an offline phase, we think it is a promising method due to the development of SLAM technology. Xu el al. [53] proposed a novel monocular visual method to locate positions in offices based on ceilings. This strategy may not function in large indoor scenes like museums or airports because these places usually do not have a planar ceiling floor with a series of chessboard-like blocks like offices. Our solution to locate users is more workable in large indoor environments. Static objects such as doors and windows are not only common in indoor scenes, but also relatively large enough to be captured from a long distance. The longest distance in our experiment is nearly 40 m, with a positioning accuracy of 0.7 m.

There are some factors that may influence the performance of our system: illumination of the indoor scenes as well as shooting angles may change images and have an impact on feature matching. In addition, the performance of the smartphones may also affect the final result. The system does not consume much battery power of smartphones as our system is web-based and the smartphone only take images as input. The clarity of images taken by smartphone cameras is high enough for our task. The distortion of images by different smartphones may change the final position output, but a camera calibration process can fix this problem (details can be found in our previous research [5]). In the future, we will try to improve the robustness of our system and experiment in other large indoor spaces with more rooms and more complex topologies.

## 6. Conclusions

In this paper, a positioning system in large indoor spaces by using smartphone cameras based on static objects is proposed. Our system uses smartphone images to detect specific static objects indoors and calculate users’ position. The system imitates the human brain’s cognitive mode and integrates algorithms of deep learning and computer vision. We experimented in an art museum with a large indoor area and a complex visual environment. Experimental results show that this method has the ability to achieve the positioning accuracy within 1 m in a distance range of 40 m indoors. We believe that it has potential for wide application in large indoor scenes.

## Figures and Tables

**Table 1:** Performance of Faster-RCNN network to detect and identify static objects. The ground truth of test images is offered by human eye judgement.

Phase	Static Object	Accurate Precision (AP)
**Training**	door1	100%
	door2	100%
	door3	90.9%
	mean	97.0%
**Testing**	door1/door2/door3	100%
